# Factors associated with willingness to take up indoor residual spraying to prevent malaria in Tororo district, Uganda: a cross-sectional study

**DOI:** 10.1186/s12936-017-2163-7

**Published:** 2018-01-05

**Authors:** Ignatius Wadunde, Arthur Mpimbaza, David Musoke, John C. Ssempebwa, Michael Ediau, Doreen Tuhebwe, Yeka Adoke, Rhoda K. Wanyenze

**Affiliations:** 1 Department of Health Policy, Planning and Management, School of Public Health, Makerere University College of Health Sciences, P.O. Box 7072, Kampala, Uganda; 2 Child Health and Development Centre, School of Medicine, Makerere University College of Health Sciences, P.O. Box 6717, Kampala, Uganda; 3 Department of Disease Control and Environmental Health, School of Public Health, Makerere University College of Health Sciences, P.O. Box 7072, Kampala, Uganda; 4 Infectious Diseases Research Collaboration, P.O. Box 7475, Kampala, Uganda

**Keywords:** Indoor residual spraying, Malaria prevention

## Abstract

**Background:**

Indoor residual spraying (IRS) is an efficient method of preventing malaria in homes, and community willingness to take up IRS is critical to its success. The first phase of IRS was conducted in Tororo district, Uganda between December 2014 and January 2015. High coverage rates (90%) were attained in the district. However, Mulanda sub-county had the lowest coverage of 78%, in the first round. This study assessed willingness and associated factors of IRS uptake among household heads for the next IRS campaign in Mulanda sub-county, Tororo district.

**Methods:**

A household survey was conducted in all three parishes of Mulanda sub-county. A multistage sampling technique involving the village and household as the first and second sampling levels, respectively, was used to identify 640 households Household heads were interviewed using standard questionnaire. Seven key informants were also conducted to explore the impact of community IRS-perceptions on uptake. Bi-variable and multi-variable logistic regression analyses were used to identify factors associated with willingness to take up IRS. Qualitative data was analysed by thematic content analysis method.

**Results:**

Most (79.9%) respondents were willing to take up repeat IRS. However this was below the target of 85%. Fear of insecticide adverse effects (62%) was the most common reason mentioned by 134 (21%) household heads who were not willing to take up IRS. Factors associated with to take up IRS were; age ≥ 35 years (AOR 1.9; 95% CI 1.08–3.51), higher socio-economic status (AOR 0.4; 95% CI 0.27–0.98), not taking IRS in previous round (AOR 0.1; 95% CI 0.06–0.23), not knowing reason for conducting IRS (AOR 0.4; 95% CI 0.24–0.78) and having an iron sheet roof (AOR 2.2; 95% CI 1.03–4.73). Community and religious leaders were the preferred sources of IRS information.

**Conclusions:**

The level of willingness to take up IRS was low (79%) compared to the targeted 85%. Involvement of community and religious leaders in community sensitization on the efficacy and safety of the chemicals could increase uptake of IRS.

## Background

Globally, malaria is a major public health problem [1] and in Africa, a child dies every minute from the disease [2]. In Uganda, malaria remains the leading cause of mortality and morbidity. Malaria is responsible for 21% of all hospital deaths in Uganda [3] and currently, 95% of the country’s population is at risk of getting malaria [4].

The World Health Organization (WHO) recommends the use of indoor residual spraying (IRS) [5–7], as a proven and highly effective malaria control measure, which involves the spraying of residual insecticide on the interior walls of homes to kill mosquitoes and interrupt malaria transmission [8–10]. Several countries in sub-Saharan Africa, including Uganda, have added IRS to their malaria control plans [11], in line with the Global Malaria Action Plan launched by the WHO Roll Back Malaria Partnership [12]. Community protection is conferred by IRS when at least 85% of houses in a targeted area are sprayed [13, 14]. However, IRS remains underutilized in sub-Saharan Africa [15].

The success of IRS relies a lot on how the benefitting communities view and embrace it [16–19]. Individual factors like age, sex, level of education, marital status, religion and occupation of household head are associated with willingness to take up IRS [20–22]. Other factors include house floor, roof and wall material [23], knowledge of IRS and its outcomes, the source of IRS information, fear of harmful health effects of IRS [18, 20, 21, 24] and preferred malaria prevention methods [8, 25].

Tororo is a malaria endemic district [26, 27]. The government of Uganda under the Malaria Control Programme (MCP) implemented the first phase of the IRS programme in Tororo between December 2014 and January 2015, and the second phase in June–July 2015. Although the district achieved 90% coverage in the first phase, there were a lot of community concerns about the IRS health risks. Mulanda sub-county had the lowest coverage of 78% in the first phase of the IRS [28]. Despite the government under the Ministry of Health and partners in Malaria Control Programme undertaking targeted social and behavioural change communications like community sensitization using health workers, community and religious leaders, village health teams and radios [28], there were community concerns about the IRS spraying like; the insecticides used could contaminate food in the houses and could cause health risks and difficulty in removing household properties, among others. This suggested that the uptake of the intervention in the second phase could be a challenge.

This study was designed to assess community willingness to take up IRS in the next round and associated factors, in Mulanda sub-county, Tororo district, to inform improvements in subsequent rounds.

## Methods

This study was a descriptive cross sectional household survey employing both qualitative and quantitative methods of data collection.

### Study area

The study was conducted in Mulanda sub-county, Tororo district, Uganda between May and June 2015 before the second round of IRS spray. The sub-county has three parishes of Mwelo, Lwala and Mulanda. Overall, the sub county has 104 villages and 9546 households. There are 36 villages in Mwelo and 34 in Mulanda and Lwala sub-counties. Agriculture is the backbone of the district economy and most of the district produce is consumed locally or sold in the urban areas within the district [29].

### Sampling procedure and sample size determination

This study, since it was a cross sectional study, the sample size (n) was determined from the formula for estimating sample sizes for prevalence studies. Therefore, the study used the formula for single proportion to calculate the number of respondents (household heads) interviewed in this cross sectional study [30] that is (Z^2^ p (1 − p))/d^2^, where, n = sample size, d = precision/error: a precision of 5% was used, Z = standard normal deviation corresponding to 95% confidence interval (1.96), p = 50% since there was no information on willingness to take up IRS in Tororo district; the study used a p = 50%.

Thus, sample size n = (1.96^2^ × 0.5 × 0.5) ÷ (0.05^2^), n = 384.

Adjusting for design effect of 1.5%, the adjusted sample N was, (N * 1.5) N = (384 * 1.5), n = 576.

Adding 10% non-response rate, N = (n/1 − 0.1), N = (576/1 − 0.1), N = 640.

The calculated sample size was 640 household heads.

The study was conducted among 640 households in Mulanda sub county, Tororo district. All household heads aged 18 years and above or emancipated minors or their designate who provided informed consent were included in the study. This sub county was deliberately selected since it had the lowest IRS coverage [28]. Mulanda sub county has three parishes of Lwala, Mulanda, and Mwelo. These three parishes had a total of 104 villages, with Mulanda parish having 36, Mwelo 34 same as Lwala with 34. A list of villages in each parish was obtained from the planning department of Tororo district office. Fifty percent (52/104) of the villages in each of the three parishes in Mulanda sub-county were selected using probability proportionate to size sampling. Thus, 17/34 villages were sampled from the parishes of Lwala and Mulanda and 18/36 from Mwelo parish.

The study used probability proportionate to size sampling to determine the total number of households to be selected in each parish. This was determined by dividing the total number of sampled villages in that particular parish (Mulanda was 17, Mwelo was 18 and Lwala was 17), by the total number of sampled villages in all the three parishes which was 52, then multiplied by 640 the overall sample size. The study thus sampled 209 households from Mulanda, 222 from Mwelo and 209 from Lwala sub counties.

The number of households to be visited in each village was determined by dividing the village size (total number of households in that village) by the total population (overall total number of households) in all the 52 selected villages which was 3812, then multiplied by 640 which was the overall sample size. To determine the exact households whose household heads were to be interviewed, the study used systematic sampling procedure. A sampling interval was computed by dividing the total number of households in the village by the required sample of households for the study in that village. The first household to be visited was randomly selected from the list of households for the respective villages using a table of random numbers [20]. The subsequent households were selected systematically from the village list of households using the calculated sampling intervals for the respective villages, until the sample size for that village was achieved. If the household head was not available, the spouse or other household member above 18 years was interviewed. In this study, all the respondents consented and were all interviewed.

### Data collection

Quantitative data was collected using a structured interviewer administered questionnaire and qualitative data by an open ended key informant guide. The structured questionnaire assessed household head individual factors, enabling and health system factors associated with willingness to take up IRS in Mulanda sub-county, Tororo district. The authors conducted both the structured and key informant interviews. Seven key informants were interviewed, including the malaria focal person at the district health office, the spray operators, village health teams and local council officials of Mulanda sub-county. The key informants were selected basing on their knowledge of indoor residual praying and their experience with factors associated with community willingness to take up IRS. The informants were interviewed in the language they were comfortable with, at their homes.

### Data management and analysis

Data entry and validation was done in EPI info statistical software and exported to Stata software for analysis. The outcome variable was “willingness to take up IRS”, and was defined as a household head willing to allow spray operators to enter and spray their houses with residual chemicals that kill mosquitoes, to prevent malaria. It was a categorical variable with yes and no categories. The independent predictors included individual level and health system factors. The enabling factors included the household heads belief that IRS is useful, the perceived benefits of IRS and perceived threats of IRS, among others. These factors were categorical variables and were analysed as frequencies and proportions. Individual factors included household heads’ gender, occupation, level of education, religion, residence, the house roof, wall and floor materials, type and size of the family, if the household head has ever heard of IRS, knowledge of the chemical used in IRS, time and frequency of spraying, among others. These were also categorical variables analysed using frequencies and proportions. Age of the household head was a continuous variable analysed using means and standard deviation. Health system factors such as distance from health facility, if a member of the household had ever suffered from malaria among others, were also analysed using frequencies and proportions.

Uni-variable analysis was done to describe the characteristics of the respondents and households. Categorical variables were analysed using frequency and proportion and continuous variables using means and standard deviations. Willingness to take up IRS was calculated by dividing the number of respondents, who reported intention to allow spray operators to enter their living houses to spray using residual chemicals to prevent malaria in the second round of spraying, by the total number of respondents interviewed.

Respondents who were aware of IRS were determined by asking whether one had ever heard of IRS or not, knowledge of the chemical used in IRS, knowledge of the exact part of the house to be sprayed, and knowledge of the time of spraying and knowledge of the frequency of spraying. Respondents’ knowledge was categorized as knowledgeable or not knowledgeable about IRS. A respondent was considered knowledgeable about IRS if he/she had ever had of IRS, knew the part of house sprayed, the time and frequency of spraying. One was considered not knowledgeable if he/she had never had of IRS, even if he/she knows the part of house to be sprayed, the time and frequency of spray. This was adapted from a similar study conducted in Soroti district assessing community knowledge about IRS [20].

Indicators that were used to develop the wealth quintiles included household possessions such as ownership of a television, mobile phone, sofa set, bed, land, livestock and permanent house; which were used as a proxy for wealth. Each categorical asset was assigned a score of 1 or 0 depending on weather the household owned that asset or not. Categorical variables were transformed into separate dichotomous (0–1) indicators. These indicators were then examined using a principal components analysis to produce a common factor score for each household [20].

Bi-variable analysis was done to determine factors associated with willingness to take up IRS. A p value corresponding to a 95% confidence interval of less than 0.05 was taken as a statistically significant association.

Multivariable logistic regression was done by backward elimination method on all variables that were found to be significant after bi-variable analyses, to identify factors independently associated with willingness to take up IRS. Variables that had a p value < 0.2 were included in the multi-variable model, to cater for confounding. The association of predictor variables with the dependent variable was described using 95% confidence interval. A p value of < 0.05 was considered statistically significant. Qualitative data was analysed by thematic content analysis method. The data was manually summarized into meaningful categories and themes and presented as quotes [23].

## Results

### Socio-demographic characteristics of the study participants

A total of 640 individuals were interviewed from 640 households. The mean age of the respondents was 38.6 years [standard deviation (SD) 12]. Over three quarters, 76% (486/640) of the respondents were males and 77.9% (499/640) were married. Majority 89% (567/640) of the respondents had attained at least primary level education. Over half of the respondents, 52% (330/640) were Anglican, majority of the respondents 90% (575/640) were farmer and all the respondents had an equal mix of the rich, middle class and the poor socio-economic class (Table 1).

**Table 1:** Socio-demographic characteristics of the study respondents

Variable	Frequency (n)	Percentage (%)
Age		
≤ 22	110	17.2
> 22	530	82.8
Gender		
Male	486	75.9
Female	154	24.1
Parish		
Lwala	292	45.6
Mulanda	154	24.1
Mwelo	194	30.3
Marital status		
Single	70	10.9
Married	499	77.9
Widowed	71	11.1
Religion		
Anglican	330	51.6
Catholic	250	39.1
Moslem	9	1.4
Pentecostal	51	7.9
Education		
None	73	11.4
Primary	461	72.0
Secondary	88	13.7
Tertiary	18	2.8
Occupation		
Farmer	575	89.8
Others	65	10.2
Wealth status		
Poor	195	30.5
Middle	221	34.5
Rich	224	35.0

### Uptake of IRS in the first round of spray

In the first round of spraying, most, 77.5% (496/640) of the respondents had their houses sprayed. Among the respondents whose houses were not sprayed in the first round, 43% (62/144) were not at home at the time of the spraying, while the rest, 57% (82/144) feared the health side effects that could result from the chemicals used in IRS. Results from qualitative data also highlighted fears of negative health effects due to chemical used in IRS.

*“During the first round of spray, there were households in my community that were not sprayed. The household heads refused us to spray there houses, claiming the chemical being used causes cancer, may kill their domestic animals and may spoil their food. The community members were also scared of the future side effects which in the long run would affect them and their children.”*

(Spray operator).

*“Some houses were closed and occupants were not at home during the time of house spraying, when we came back the following day, the household members’ claimed they were in the gardens far from their homes. We also realized that they just did not want their houses to be sprayed because they did not allow us to enter and spray after all, even when they knew that we were to come and spray their houses.”*

(Spray operator).

### Willingness to take up IRS in the next round of spray

Most, 79% (506/640) of the respondents were willing to take up IRS in the next round of spray. Among the reasons for conducting IRS that the respondents mentioned were to kill mosquitoes, 51.4% (329/640) and to kill other insects in the houses, 23% (148/640). A quarter of the respondents, 26% (163/640) were not sure of the use of IRS. The respondents who were not willing to take IRS in the next round of spray, 21% (134/640) gave the reasons that the first round of spray did not work, 38% (51/134), while the rest, 62%, (83/134) feared health effects that may result from the residual chemicals used in IRS.

The most preferred method of receiving IRS information was the use of community leaders, 81.6% (522/640). A few of the respondents, 6% (39/640) preferred health workers while slightly more, 12.4% (79/640) preferred religious leaders.

Key informants agreed that the most preferred channel of passing out IRS information to the communities, by community members is through local community and religious leaders. This can be illustrated by the KI quotations below.

*“I think the best way of passing out IRS information to the communities is by the local leaders addressing communities in public functions like burials. It would also be better to use institutional leaders like in schools, churches and cultural institutions.”*

(KI: Village Health Team).

### Bi-variable analysis

#### Individual factors associated with willingness to take up IRS

Age, residence, religion, wealth status and the type of roof materials were associated with willingness to take up IRS in the next round of spray. Respondents who had primary education and higher were more willing to take up IRS in the next round compared those who had no formal education (OR = 3.6, 95% CI 1.81–7.04). Older respondents (above 22 years) were more likely to take up IRS in the next round of spray compared to those younger than 22 years (OR 4.6, 95% CI 2.99–7.24). Compared to the poor, the middle and higher socio-economic class were less likely to take up IRS in the next round of spray (OR 0.6, 95% CI 0.34–0.94). Respondents who had houses roofed with iron sheets were more likely (OR 1.5, 95% CI 1.04–2.24) to take up IRS in next round compared to those with grass thatched houses.

All (100%) respondents reported to have heard about IRS. Respondents whose houses were not sprayed in round 1 were less likely to take up IRS in the next round of spray (OR 0.2, 95% CI 0.09–0.27). Respondents who did not know the reason for conducting IRS (as to kill mosquitoes) were less likely to take up IRS in the next round of spray (OR 0.4, 95% CI 0.26–0.76). Compared to respondents who were knowledgeable about IRS, respondents who were not knowledgeable were less likely to take up IRS in the next round of spray (OR 0.4, 95% CI 0.27–0.65). Respondents who knew the reason for spraying (to kill mosquitoes) and knew the frequency of spraying (after every 6 months) were considered knowledgeable about IRS (Table 2).

**Table 2:** Individual factors associated with willingness to take up IRS in next round

Variable	Willingness to take up IRS in next round		COR	95% CI	p value
	No (%)	Yes (%)			
Level of education					
None	32 (23.9)	41 (8.1)	1		
Primary	83 (61.9)	378 (74.7)	3.5	(2.11–5.98)	< 0.001^a^
Secondary and above	19 (14.2)	87 (17.2)	3.6	(1.81–7.04)	< 0.001^a^
Age					
≤ 22	50 (37.8)	60 (11.8)	1		
> 22	83 (62.4)	447 (88.2)	4.6	(2.99–7.24)	< 0.001^a^
Sex					
Male	98 (73.1)	388 (76.7)	1		
Female	36 (26.9)	118 (23.3)	0.8	(0.54–1.28)	0.394
Occupation					
Farmer	120 (89.5)	455 (89.9)	1		
Others	14 (10.5)	51 (10.1)	0.9	(0.51–1.79)	0.900
Residence (parish)					
Mulanda	21 (15.7)	133 (26.3)	1		
Lwala	75 (56.0)	217 (42.9)	0.4	(0.27–0.78)	0.004^a^
Mwelo	38 (28.4)	156 (30.8)	0.6	(0.36–1.16)	0.144
Religion					
Anglican	77 (57.5)	253 (50.0)	1		
Catholic	35 (26.1)	215 (42.5)	1.8	(1.21–2.89)	0.005^a^
Others	22 (16.4)	38 (7.5)	0.5	(0.29–0.94)	0.031^a^
Wealth status					
Poor	27 (20.3)	168 (33.1)	1		
Middle	49 (36.8)	172 (33.9)	0.6	(0.34–0.94)	0.030^a^
Rich	57 (42.9)	167 (32.9)	0.5	(0.28–0.28)	0.003^a^
Family type					
Monogamous	113 (89.0)	402 (86.3)	1		
Polygamous	14 (11.0)	64 (13.7)	1.3	(0.69–2.38)	0.424
Family size					
≤ 5 members	99 (73.9)	364 (71.9)	1		
More than 5 members	35 (26.1)	142 (28.1)	1.1	(0.71–1.69)	0.655
House floor material					
Natural floor (dung)	118 (88.1)	452 (89.3)	1		
Finished floor (cemented)	16 (11.9)	54 (10.7)	0.9	(0.49–1.59)	0.676
House roof material					
Natural roof (thatched)	73 (54.9)	222 (43.9)	1		
Finished (iron sheets)	61 (45.1)	284 (56.1)	1.5	(1.04–2.24)	0.029^a^
House wall material					
Natural walls	84 (62.7)	289 (57.1)	1		
Finished (bricks, cement)	50 (37.3)	217 (42.9)	1.3	(0.85–1.87)	0.245
Took up IRS in first round					
Yes	98 (73.1)	478 (94.5)	1		
No	36 (26.9)	28 (5.5)	0.2	(0.09–0.27)	< 0.001^a^
Source of information about IRS					
Community leaders	102 (761)	420 (83.0)	1		
Health workers	20 (14.9)	59 (11.7)	0.5	(0.27–1.14)	0.097
Others	12 (8.9)	27 (5.3)	0.7	(0.41–1.24)	0.236
Knew the chemical used in IRS					
Yes	11 (8.3)	27 (5.3)	1		
No	121 (91.7)	478 (94.7)	1.6	(0.78–3.33)	0.201
Knew frequency of spraying					
Yes	90 (67.2)	363 (71.5)	1		
No	44 (32.8)	144 (28.5)	0.3	(0.28–0.46)	< 0.001^a^
Reason for spraying					
To kill mosquitoes	32 (23.9)	297 (58.7)	1		
To kill other insects	29 (21.6)	119 (23.5)	0.4	(0.26–0.76)	0.003^a^
Don’t know	73 (54.5)	90 (17.8)	0.1	(0.08–0.21)	< 0.001^a^
Knowledge of IRS					
Knowledgeable	31 (23.1)	212 (49.9)	1		
Not knowledgeable	103 (76.9)	294 (58.1)	0.4	(0.27–0.65)	< 0.001^a^

^a^Statistically significant

Results from qualitative data shows that all the communities of Tororo district were aware of the re-spraying of houses after the first round of spray. The awareness was created through use of local radios and community and religious leaders.

*“We used local leaders, religious and cultural leaders in the communities to tell people in about the re*-*spraying phases of IRS. I am sure everyone is aware that after six months, the spray operators would come back to spray their houses.”*

(KI: Malaria focal person).

The KIs agreed that there were still information gaps about IRS in the communities. The information gaps included; not knowing the effectiveness of the chemicals used in IRS, why the spray is done only in the inside walls of houses and why they do not spray the latrines and bathrooms. The KIs noticed this information gap about IRS could lead to reduced uptake of IRS in the subsequent phases of sprays.

*“Some people now fall back and refuse their houses to be sprayed because they wonder why when houses are sprayed, if a hen eats a dead cockroach that died due to the spray chemical, the hen also dies.”*

(KI: Village Health Team).

The KIs also agreed that there is need for addressing this knowledge gap about IRS through continued sensitizations using community leaders, so as to ensure increased uptake of IRS in the subsequent rounds of spray.

*“There is need for more sensitization of communities using community and church leaders. There is also need of dialoging with the communities to using local councils, village health teams and church leaders. This is because these people are respected in the communities”*

(Malaria focal person).

#### Enabling factors associated with willingness to take up IRS

The enabling factors were based on how the respondents perceived the benefits and threats of taking up IRS. Respondents who believed that IRS does not reduce mosquitoes were less likely to take it up in the next round of spray compared to those who agreed (OR 0.3, 95% CI 0.19–0.42). Similarly, compared to respondents who agreed that IRS reduces chances of getting malaria, those who disagreed were less likely to take it up in the next round of spray (OR 0.3, 95% CI 0.22–0.46). Respondents who disagreed that IRS has negative health effects were more likely to take up IRS in the next round of spray compared to those who agreed (OR 1.9, 95% CI 1.52–2.41). Similarly, compared to the respondents who believed that the chemicals used will pollute the environment, those who disagreed were two times likely to take it up in the next round of spray (OR 2.0, 95% CI 1.55–2.41). Respondents who perceived IRS as not useful were less likely to take it up in the next round of spray compared to those that perceived it as useful (OR 0.1, 95% CI 0.04–0.18) (Table 3).

**Table 3:** Enabling factors associated with willingness to take up IRS in next round

Variable	Willingness to take up IRS in next round		COR	95% CI	p value
	No (%)	Yes (%)			
Believed that IRS reduces nuisance of mosquitoes					
Agree	109 (81.3)	497 (98.2)	1		
Disagree	25 (18.7)	9 (1.8)	0.3	(0.19–0.42)	< 0.001^a^
Believed that IRS reduces chances of getting malaria					
Agree	110 (82.1)	495 (97.8)	1		
Disagree	24 (17.9)	11 (2.2)	0.3	(0.22–0.46)	< 0.001^a^
Believed that IRS reduces chances of getting malaria					
Agree	42 (31.8)	57 (11.3)	1		
Disagree	90 (68.2)	449 (88.7)	1.9	(1.52–2.41)	< 0.001^a^
Believed that chemicals used in IRS pollute environment					
Agree	30 (22.4)	33 (6.5)	1		
Disagree	104 (77.6)	473 (93.5)	2.0	(1.55–2.66)	< 0.001^a^
Had children under 5 years					
Yes	75 (53.0)	242 (47.9)	1		
No	63 (47.0)	263 (52.1)	1.2	(0.84–1.79)	0.298
Preferred method of preventing malaria					
IRS	7 (5.2)	31 (6.1)	1		
ITN	127 (94.8)	475 (93.9)	0.8	(0.36–1.96)	0.695
Believed that IRS is useful					
Useful	61 (45.5)	314 (62.1)	1		
Fairly useful	49 (36.6)	182 (35.9)	0.7	(0.47–1.09)	0.126
Not useful	24 (17.9)	10 (2.0)	0.1	(0.04–0.18)	< 0.001^a^
Household had a pregnant woman					
Yes	12 (9.0)	32 (6.3)	1		
No	122 (91.0)	474 (93.7)	1.4	(0.73–2.91)	0.287
Member of household had malaria a month prior interview					
Yes	64 (47.8)	240 (47.6)	1		
No	70 (52.2)	264 (52.4)	1.0	(0.69–1.47)	0.977
Source of malaria treatment					
VHT	15 (22.4)	29 (12.2)	1		
Public health center	42 (62.7)	177 (74.7)	2.2	(1.07–4.43)	0.031^a^
Private health center	10 (14.9)	31 (13.1)	1.6	(0.62–4.13)	0.328

^a^Statistically significant

The KIs agreed that, despite the fact that most community members perceived IRS as beneficial some (including those that perceived IRS as useful) perceived it as harmful anyway. The likely negative perceptions about IRS were the fears of the health side effects of the chemicals used in IRS.

*“Initially there was a lot of fear about IRS in our communities following what people suffered in northern Uganda after the chemicals were sprayed nodding disease started.”*

(KI: Community leader).

The KIs again agreed that the negative perceptions about IRS will affect community willingness to take up the intervention in the next coming rounds and that there is need to address the communities about the negative perceptions about IRS through community dialogues, sensitizations using village health teams, community and religious leaders.

*“The negative perceptions about IRS could affect community willingness to take up IRS in next rounds of spray. There is need to address the communities about these negative perceptions about IRS through community dialogues using village health teams, local NGO’s and CBO’s, community and local leaders to ensure all household accept a their houses to be sprayed in the next round.”*

(KI: Malaria focal person).

#### Health systems factors associated with willingness to take up IRS

Compared to respondents who walked to the nearest health facility, those who used a bicycle or motor cycle were less likely to take up IRS in the next round of spray (OR 0.4, 95% CI 0.39–0.68). Respondents of households that had more than two mosquito nets were less likely to take up IRS in the next round of spray compared to those that had two or less (OR 0.6, 95% CI 0.38–0.83) (Table 4).

**Table 4:** Health system factors associated with willingness to take up IRS in the next round

Variable	Willingness to take up IRS in next round		COR	95% CI	p value
	No (%)	Yes (%)			
Distance to nearest health centre					
≤ 5 km	129 (96.3)	469 (92.7)	1		
> 5 km	5 (3.7)	37 (7.3)	0.1	(0.78–5.28)	0.144
Means of transport to health centre					
Walking	85 (63.4)	442 (87.4)	1		
Bicycle	23 (17.2)	47 (9.3)	0.4	(0.39–0.68)	0.001^a^
Other means	26 (19.4)	17 (3.36)	0.2	(0.06–0.24)	< 0.001^a^
Had a community medicine distributor					
Yes	91 (67.9)	363 (71.7)	1		
No	36 (26.9)	115 (22.7)	0.8	(0.52–1.24)	0.322
Don’t know/not sure	7 (5.2)	28 (5.5)	1.0	(0.42–2.37)	0.995
Number of mosquito nets in a household					
Two or less	55 (41.0)	280 (55.3)	1		
More than two	79.9 (58.9)	226 (44.7)	0.6	(0.38–0.83)	0.003^a^
Source of IRS information					
Community leaders	102 (77.3)	420 (83.2)	1		
Others	30 (22.3)	85 (16.8)	0.7	(0.43–1.09)	0.118

^a^Statistically significant

The KIs agreed that the district and the IRS implementing agency prepared awareness activities prior the IRS, to enhance uptake of the intervention among the communities. Most of the awareness activities included; community sensitizations through the local radios, training of spray operators and community mobilizations through community and religious leaders. The KIs also said that the most preferred channel of passing out IRS information to the communities is the use of community and religious leaders. This can be illustrated by the KI response below.

“*The district, after the first round of spray, conducted dissemination exercises to review performance of every sub county. The district went ahead and gathered feedback from the exercise of what transpired, lessons learned and how to improve uptake in the subsequent sprays. There is need for community and religious leaders to address communities during public gatherings like burials and marriage ceremonies.”*

(KI: Sub-county chief).

### Multivariable analysis

After controlling for potential confounders, age, having taken up IRS in the first round, wealth status, knowing the reason for conducting IRS and the type of house roof were independently associated with willingness to take up IRS in the next round of spray. Compared to younger respondents (≤ 22 years), older ones (> 35 years) were approximately two times likely to take IRS in the next round of spray (AOR 1.9, 95% CI 1.08–3.51). The middle socio-economic class respondents were less likely to take up IRS in the next round of spray compared their poor counterparts (AOR 0.4, 95% CI 0.27–0.98). The odds of willingness to take up IRS in the next round of spray in the respondents who did not take up IRS in previous round was reduced (AOR 0.1, 95% CI 0.06–0.23). Respondents who believed that IRS does not kill mosquitoes were less likely to take IRS in the next round of spray compared to their counterparts who believed that IRS kills mosquitoes and reduces chances of getting malaria (AOR 0.4, 95% CI 0.24–0.78). Respondents whose households had houses with an iron sheet roof were more likely to take up IRS in the next round of spray compared to those that their houses were thatched (AOR 2.2, 95% CI 1.03–4.73) (Table 5).

**Table 5:** Multivariable analysis of factors associated with willingness to take up IRS in next round

Variable	UOR			95% CI	p value	AOR	95% CI		p value
Level of education									
None	1					1			
Primary	3.5			(2.11–5.98)	< 0.001^a^	1.4	(0.69–2.73)		0.362
Secondary and above	3.6			(1.81–7.04)	< 0.001^a^	1.5	(0.66–3.56)		0.316
Age									
≤ 22	1					1			
23–35	1.5			(0.89–2.59)	0.126	1.3	(0.72–2.46)		0.361
> 35	1.9			(1.19–3.12)	0.007	1.9	(1.08–3.51)		0.026^a^
Residence (parish)									
Mulanda	1					1			
Lwala	0.4			(0.27–0.78)	0.004^a^	0.8	(0.45–1.42)		0.443
Mwelo	0.6			(0.36–1.16)	0.144	1.2	(0.63–2.27)		0.592
Religion									
Anglican	1					1			
Catholic	1.8			(1.21–2.89)	0.005^a^	1.0	(0.65–1.67)		0.863
Others	0.5			(0.29–0.94)	0.031^a^	1.4	(0.64–3.04)		0.396
Wealth status									
Poor	1					1			
Middle	0.6			(0.34–0.94)	0.030^a^	0.5	(0.27–0.98)		0.043^a^
Rich	0.5			(0.28–0.28)	0.003^a^	0.4	(0.17–1.14)		0.091
Source of IRS information									
Community leaders	1					1			
Health workers	0.5			(0.27–1.14)	0.097	0.8	(0.32–1.90)		0.580
Others	0.7			(0.41–1.24)	0.236	0.7	(0.33–1.41)		0.303
Took IRS in first round									
Yes	1					1			
No	0.2			(0.09–0.27)	< 0.001^a^	0.1	(0.06–0.23)		<0.001^a^
Knew frequency of spraying									
Yes	1					1			
No	0.3			(0.28–0.46)	< 0.001^a^	0.9	(0.55–1.51)		0.714
Reason for conducting IRS									
To kill mosquitoes	1					1			
To kill other insects	0.4			(0.26–0.76)	0.003^a^	0.4	(0.24–0.78)		0.005^a^
Don’t know	0.1			(0.08–0.21)	< 0.001^a^	0.2	(0.12–0.35)		< 0.001^a^
Believed IRS has health effects									
Agree	1					1			
Disagree	1.9			(1.52–2.41)	< 0.001	1.1	(0.65–1.73)		0.815
Believed chemicals used will pollute the environment									
Agree	1					1			
Disagree	2.0			(1.55–2.66)	< 0.001	1.2	(0.69–2.16)		0.482
Number of mosquito nets in the household									
Two or less	1					1			
More than two	0.6			(0.38–0.83)	0.003^a^	0.82	(0.52–1.30)		0.404
House roof materials									
Grass thatched	1					1			
Iron sheets	1.5			(1.04–2.24)	0.029^a^	2.2	(1.03–4.73)		0.042^a^

^a^Statistically significant

The relationship between level of education and willingness to take up IRS was confounded by age; there was no effect modification since the different strata of level of education was almost the same. The relationship did not significantly change by levels of education.

## Discussion

This study found an overall uptake of IRS in the first round of spray of 77.5% in Mulanda sub-county Tororo district, below the 85% target of the Uganda malaria reduction strategic plan 2014–2020 [14]. Of the 144 respondents who did not have their houses sprayed, 43% (62/144) were not at home, while the rest, 59.6% (82/144) feared health side effects of the chemicals, a pointer to the gaps in community preparedness. However, the uptake of IRS in this community is higher than that reported in Mozambique [18].

The proportion of respondents who were willing to take up IRS in the next round of spray was 79%, slightly higher than the 77.5% in the first round. This is a concern given that the 79% willingness may not result into actual uptake if absence of family members of targeted households and other issues come into play. This willing to take up IRS in the next round of spray is much lower than that reported in Rakai, where over 90% of the respondents were willing to take IRS in their homes [21]. Addressing the concerns about lack of efficacy and fears of health effects could further increase the willingness and uptake [22].

In this study, respondents who were above 35 years were more willing to take up IRS in the next round compared to their younger counterparts. These findings are similar to those found in a study in Eastern Uganda [20] and could be attributed to lived experience and their ability to view IRS as important in malaria control. IRS sensitization campaigns should consider use of platforms that attract younger age groups e.g. use of community sports galas and public address systems.

The wealthier respondents were less likely to take up IRS in the next round probably because they are capable of using other malaria control methods, such as mosquito nets, which usually come at a cost. Respondents who did not take up IRS in the first round of spray were less likely to take it up in the next round probably because the same concerns about health effects may have hindered uptake and still persist. It is thus not surprising that those who did not know the reason for spraying were less likely to take up IRS in the next round [21] and emphasizing the need to address these issues in community education.

This study assessed willingness to take up repeat IRS, which may not necessarily reflect actual uptake. However, the study highlights knowledge gaps and fears of health effects which may hinder uptake of repeat screening, if not addressed. The authors also noted that it would be great to include a control population where there was a higher coverage of IRS, so as better detect the key factors responsible of the low coverage.

## Conclusions

The level of willingness to take up IRS was sub optimal (79%) compared to the targeted 85% by 2015. There is need for community mobilization and sensitization campaigns to ensure that communities appreciate the reason for conducting IRS, the efficacy and safety of the chemicals used in IRS. Platforms that can attract younger people and use of religious and community leaders could be helpful in reaching out to those who still have knowledge gaps and misconceptions about IRS.

## Abbreviations

CDC: Centers for disease control and prevention
IRS: indoor residual spraying
MaKSPH: Makerere University School of Public Health
MCP: Malaria Control Programme
MOH: Ministry of Health
VHT: Village Health Team
WHO: World Health Organization
